# Alpha‐synuclein dynamics in induced pluripotent stem cell‐derived dopaminergic neurons from a Parkinson’s disease patient (*PARK4*) with *SNCA* triplication

**DOI:** 10.1002/2211-5463.13060

**Published:** 2021-01-05

**Authors:** Hayato Fukusumi, Kazuyuki Togo, Miho Sumida, Masayuki Nakamori, Satoshi Obika, Kousuke Baba, Tomoko Shofuda, Daisuke Ito, Hideyuki Okano, Hideki Mochizuki, Yonehiro Kanemura

**Affiliations:** ^1^ Division of Stem Cell Research Department of Biomedical Research and Innovation Institute for Clinical Research National Hospital Organization Osaka National Hospital Japan; ^2^ Department of Neurology Graduate School of Medicine Osaka University Japan; ^3^ Division of Regenerative Medicine Department of Biomedical Research and Innovation Institute for Clinical Research National Hospital Organization Osaka National Hospital Japan; ^4^ Graduate School of Pharmaceutical Sciences Osaka University Japan; ^5^ Department of Neurology Keio University School of Medicine Tokyo Japan; ^6^ Department of Physiology Keio University School of Medicine Tokyo Japan; ^7^ Department of Neurosurgery National Hospital Organization Osaka National Hospital Japan

**Keywords:** dopaminergic neuron, human‐induced pluripotent stem cell, *PARK4*, Parkinson's disease, *SNCA* triplication, α‐synuclein

## Abstract

Parkinson's disease (PD) is a neurodegenerative disorder caused by the selective loss of dopaminergic (DA) neurons in the substantia nigra pars compacta (SNc). Lewy bodies (LBs), another histological hallmark of PD, are observed in patients with familial or sporadic PD. The therapeutic potential of reducing the accumulation of α‐synuclein, a major LB component, has been investigated, but it remains unknown whether the formation of LBs results in the loss of DA neurons. *PARK4* patients exhibit multiplication of the α‐synuclein gene (*SNCA*) without any pathological mutations, but their symptoms develop relatively early. Therefore, study of *PARK4* might help elucidate the mechanism of α‐synuclein aggregation. In this study, we investigated the dynamics of α‐synuclein during the early stage of immature DA neurons, which were differentiated from human‐induced pluripotent stem cells (hiPSCs) derived from either a *PARK4* patient with *SNCA* triplication or a healthy donor. We observed increased α‐synuclein accumulation in *PARK4* hiPSC‐derived DA neurons relative to those derived from healthy donor hiPSCs. Interestingly, α‐synuclein accumulation disappeared over time in the *PARK4* patient‐derived DA neurons. Moreover, an *SNCA*‐specific antisense oligonucleotide could reduce α‐synuclein levels during the accumulation stage. These observations may help reveal the mechanisms that regulate α‐synuclein levels, which may consequently be useful in the development of new therapies for patients with sporadic or familial PD.

## Graphical abstract

Alpha‐synuclein is a major component of Lewy bodies, which are a histological hallmark of Parkinson's disease (PD). In this study, we investigated the dynamics of α‐synuclein during the early stage of immature dopaminergic neurons differentiated from human‐induced pluripotent stem cells derived from either a PD patient with *SNCA* triplication or a healthy donor.

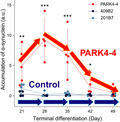

**Abbreviations**
ASO: antisense oligonucleotide
DA neuron: dopaminergic neuron
hiPSC: human‐induced pluripotent stem cell
LB: Lewy body
PD: Parkinson's disease
SNc: substantia nigra pars compacta

Parkinson's disease (PD) is a neurodegenerative disorder caused by the selective loss of dopaminergic (DA) neurons in the substantia nigra pars compacta (SNc) [1, 2]. Although studies on familial forms of PD have elucidated the chromosomal loci of causative genes (*PARK1‐23*) [3, 4], sporadic forms of PD with unknown mechanisms compose ~ 90% of PD cases [5]. Lewy bodies (LBs), which are primarily composed of α‐synuclein protein, are formed in the SNc of most patients with either familial or sporadic PD [6, 7], except for certain patients with familial PD carrying *PARK2* (*PRKN*; parkin RBR E3 ubiquitin protein ligase) and *PARK8* (*LRRK2*; leucine‐rich repeat kinase 2) [4]. Although the mechanism underlying α‐synuclein aggregation in LBs is unknown, therapies aimed at reducing α‐synuclein levels have been investigated, including the use of antisense oligonucleotides (ASOs) [8, 9] and α‐synuclein‐specific antibodies [10].

*PARK4* patients have a multiplication of the α‐synuclein gene (*SNCA*) without any pathological mutation, although their symptoms develop relatively early [11, 12]. Therefore, *PARK4* might be a useful target to investigate the mechanism through which DA neurons regulate α‐synuclein aggregation before the clinical onset of PD and could lead to the development of new therapies for patients with sporadic or familial PD.

In this study, to examine the effects of α‐synuclein aggregation on DA neurons, we investigated the dynamics of α‐synuclein in DA neurons differentiated from human‐induced pluripotent stem cells (hiPSCs) derived from a *PARK4* patient with *SNCA* triplication and a healthy donor. Furthermore, we examined the feasibility of a therapeutic strategy to reduce α‐synuclein levels using ASOs.

## Materials and methods

### Ethics statement

This study was conducted in accordance with the principles of the Declaration of Helsinki. The use of hiPSCs was approved by the ethics committee of Osaka National Hospital (approval no. 110 and 120) and Keio University School of Medicine (approval no: 20080016).

### Cell culture

The *PARK4* patient with *SNCA* triplication‐derived hiPSC line PARK4‐4 (RRID: CVCL_T870) was established from patient fibroblasts ND27760 (RRID: CVCL_F204), which were obtained from the Coriell Cell Repository, by previously described reprogramming methods [13, 14]. The written informed consent was obtained from the patient. The healthy donor‐derived hiPSC lines 201B7 (RRID: CVCL_A324) [15] and 409B2 (RRID: CVCL_K092) [16] were obtained from the RIKEN Cell Bank (Tsukuba, Japan). These cells were maintained on Corning Matrigel hESC‐qualified Matrix (cat. 354277; Corning, Corning, NY, USA) and in mTeSR1 medium (cat. 85850; STEMCELL Technologies, Vancouver, BC, Canada) supplemented with 1% antibiotic–antimycotic (Anti‐Anti; cat. 15240‐062; Thermo Fisher Scientific, Waltham, MA, USA) at 37 °C in a humidified 5% CO_2_ incubator [17].

### *SNCA* gene copy number analysis

Genomic DNA was isolated from cells using a DNeasy Blood & Tissue kit (cat. 69504; Qiagen, Hilden, Germany). The copy number of *SNCA* was determined by performing a TaqMan Copy Number assay against the 3rd intron of *SNCA* (Assay ID: Hs04791950_cn; Thermo Fisher Scientific) together with a TaqMan Copy Number Reference Assay against *TERT* (cat. 4401633; Thermo Fisher Scientific) in a duplex quantitative PCR using the TaqMan Genotyping Master Mix (cat. 4371355; Thermo Fisher Scientific) and QuantStudio 12K Flex Real‐Time PCR System (Applied Biosystems, Foster City, CA, USA). The relative copy numbers are expressed as $2^{‐\Delta \Delta C_{t}}$ values.

### Karyotype analysis

Karyotype analysis was performed by conventional Giemsa staining and G‐banding, and diagnosis was performed based on the 2013 international system for human cytogenetic nomenclature (ISCN 2013) [18].

### Differentiation of midbrain dopaminergic neurons from hiPSCs

Midbrain dopaminergic neurons (DA neuron) were differentiated from hiPSCs according to previously reported methods [19, 20], with slight modifications. On day 0, the hiPSCs were dissociated into single cells using TrypLE Select CTS (TrypLE; cat. A12859‐01; Thermo Fisher Scientific) and then were suspended in 8GMK medium containing Glasgow's MEM (cat. 11710‐035; Thermo Fisher Scientific) supplemented with 8% KnockOut Serum Replacement (cat. 10828028; Thermo Fisher Scientific), 1% MEM nonessential amino acids solution (cat. 11140‐050; Thermo Fisher Scientific), 1 mm sodium pyruvate (cat. S8636; Sigma‐Aldrich, St. Louis, MO, USA), 0.1 mm 2‐mercaptoethanol (cat. 21985023; Thermo Fisher Scientific), and 1% Anti‐Anti. Moreover, 10 µm SB431542 (SB; cat. 192‐16541; FUJIFILM Wako Pure Chemical, Osaka, Japan) and 0.1 µm LDN‐193189 (LDN; cat. Axon 1509; Axon Medchem, Groningen, the Netherlands) were added for dual SMAD inhibition [21], and 30 µm Y‐27632 (Y; cat. 036‐24023; FUJIFILM Wako Pure Chemical) was added to prevent apoptosis [22]. The cells were seeded into 96‐well plates (cat. MS‐9096V; Sumitomo Bakelite, Tokyo, Japan) at a density of 9000 cells/well in a total volume of 75 µL, and then, they were cultured at 37 °C in a humidified 5% CO_2_ incubator [23]. On day 1, 75 µL of medium [8GMK medium supplemented with dual SMAD inhibitors (10 µm SB and 0.1 µm LDN), 200 ng·mL^−1^ human SHH (C24II) (SHH; final 100 ng·mL^−1^; cat. 130‐095‐727; Miltenyi Biotec, Bergisch Gladbach, Germany), 8 µm purmorphamine (PM; final 4 µm; cat. 10009634; Cayman Chemical, Ann Arbor, MI, USA), and 200 ng·mL^−1^ recombinant human FGF‐8b (FGF8; final 100 ng·mL^−1^; cat. 100‐25; PeproTech, Rocky Hill, NJ, USA)] were added to each well (total volume: 150 µL per well). On day 3, half of the medium was replaced with 8GMK medium supplemented with 10 µm SB, 0.1 µm LDN, 100 ng·mL^−1^ SHH, 4 µm PM, 100 ng·mL^−1^ FGF8, and 6 µm CHIR99021 (CHIR; final concentration: 3 µm; cat. 13122; Cayman Chemical). On days 5 and 7, half of the medium was replaced with 8GMK medium supplemented with SMAD inhibitors (10 µm SB and 0.1 µm LDN), 100 ng·mL^−1^ SHH, 4 µm PM, 100 ng·mL^−1^ FGF8, and 3 µm CHIR. On day 9, half of the medium was replaced with 8GMK medium supplemented with 0.1 µm LDN and 3 µm CHIR. On day 11, nearly all medium was removed from each well and replaced with 75 µL of terminal differentiation medium [Neurobasal medium (NB; cat. 21103‐049; Thermo Fisher Scientific), 2% B27 supplement (B27; cat. 17504‐044; Thermo Fisher Scientific), 1% GlutaMAX‐I (cat. 35050‐061; Thermo Fisher Scientific), 1% Anti‐Anti, 20 ng·mL^−1^ brain‐derived neurotrophic factor (cat. 450‐02; PeproTech), 20 ng·mL^−1^ recombinant human glial‐derived neurotrophic factor (cat. 450‐10; PeproTech), 0.5 mm dibutyryl‐cAMP (cat. sc‐201567B; Santa Cruz Biotechnology, Santa Cruz, CA, USA), 0.2 mm ascorbic acid (cat. 03420‐52; Nacalai Tesque, Kyoto, Japan), 1 ng·mL^−1^ recombinant human TGF‐β3 (TGFβ3; cat. 100‐36E; PeproTech), and 10 µm DAPT (cat. ab120633; Abcam, Cambridge, UK) supplemented with 3 µm CHIR. On day 13, 75 µL of terminal differentiation medium supplemented with 3 µm CHIR was added to each well. On day 14, all neural aggregates that had formed were harvested and dissociated into single cells using TrypLE Select (Thermo Fisher Scientific) or Neuron Dissociation Solution (cat. 297‐78101; FUJIFILM Wako Pure Chemical). The dissociated cells were suspended in terminal differentiation medium supplemented with 30 µm Y and were cultured in 96‐well plates (cat. 89626; ibidi, Gräfelfing, Germany) at a density of 3 × 10^5^ cells per cm^2^ on Corning Growth Factor Reduced Matrigel (cat. 356230; Corning) in a volume of 100 µL. On day 16, 100 µL of terminal differentiation medium was added to each well (total 200 µL per well). After day 18, half of the medium was replaced with fresh terminal differentiation medium every other day until day 49.

### Quantitative RT‐PCR (qPCR)

For total RNA extraction, the cells were lysed in QIAzol Lysis Reagent (cat. 79306; Qiagen), and RNA was extracted using a chloroform and isopropanol precipitation method. Total RNA was then treated with Turbo DNase (cat. AM2239; Thermo Fisher Scientific) according to the manufacturer's protocol to reduce genomic DNA contamination. cDNA was synthesized from total RNA using a PrimeScript RT Reagent kit (cat. RR037A; Takara Bio, Shiga, Japan) according to the manufacturer's specifications. The cDNA was subjected to quantitative PCR analysis using gene‐specific primers/probes (Tables 1 and 2) and either PowrUp SYBR Green Master Mix (cat. A25778; Thermo Fisher Scientific) or TaqMan Gene Expression Master Mix (cat. 4369510; Thermo Fisher Scientific). All reactions were run on a 7300 Real‐Time PCR System (Applied Biosystems). Gene expression levels are expressed as ΔC*_t_* values normalized to the levels of *GAPDH* (a lower ΔC*_t_* value indicates higher expression).

**Table 1:** Primers for SYBR Green‐based quantitative PCR.

Gene	Forward primer (5′–3′)	Reverse primer (5′–3′)
*GAPDH*	CCACTTTGTCAAGCTCATTTCCT	TCTCTTCCTCTTGTGCTCTTGCT
*Oct4*	GACAGGGGGAGGGGAGGAGCTAGG	CTTCCCTCCAACCAGTTGCCCCAAAC
*NANOG*	GCAGAAGGCCTCAGCACCTA	GGTTCCCAGTCGGGTTCAC
*LIN28A*	CACGGTGCGGGCATCTG	CCTTCCATGTGCAGCTTACTC
*FOXA2*	TGCTGGTCGTTTGTTGTGG	CATGTTGCTCACGGAGGAGTAG
*LMX1A*	GATCCCTTCCGACAGGGTCTC	GGTTTCCCACTCTGGACTGC
*CORIN*	CATATCTCCATCGCCTCAGTTG	GGCAGGAGTCCATGACTGT
*TH*	GTAAGCAGAACGGGGAGGTG	GGTACGTCTGGTCTTGGTAGGG

**Table 2:** Probes for TaqMan gene expression assay.

Gene	Assay ID	Vendor
*GAPDH*	Hs03929097_g1	Thermo Fisher Scientific
*SNCA*	Hs01103383_m1	Thermo Fisher Scientific

### Determination of the OCT4/TRA‐1‐60‐positive proportion of hiPSCs

hiPSCs were dissociated into single cells using TrypLE, and then, they were fixed in a solution of 4% paraformaldehyde in phosphate buffer (cat. 163‐20145; FUJIFILM Wako Pure Chemical) for 15 min at room temperature (RT). Subsequently, the cells were washed three times with PBS. The fixed cells were suspended in a 0.1% solution of Triton X‐100 in PBS and seeded into a 96‐well plate (cat. 161093; Thermo Fisher Scientific) at a density of 2–3 × 10^4^ cells/well and allowed to settle for 10 min at RT. The 96‐well plate was then centrifuged at 300 ***g*** for 3 min at RT. The supernatant was removed carefully from each well, and the plate was dried for 10–15 min at RT. Finally, 100 µL of PBS was added to each well, and immunocytochemical staining was performed as described in the next section. The OCT4/TRA‐1‐60‐positive population was evaluated using a high‐content screening system (ArrayScan XTI HCA Reader; Thermo Fisher Scientific).

### Immunocytochemical staining (ICC)

The fixed cells were permeabilized and treated with blocking buffer containing PBS, 0.1% Triton X‐100, and 10% normal goat serum for 1 h at RT. Next, the cells were stained overnight with primary antibodies (Table 3) in blocking buffer at 4 °C. Then, the cells were washed three times with 0.01% Triton X‐100 containing PBS (TPBS) and were incubated with a solution containing secondary antibodies (Table 3) and DAPI (nuclear stain; cat. D212; Dojindo, Kumamoto, Japan) for 1 h at RT. Finally, the cells were washed three times with TPBS and were observed with a high‐content screening system or a confocal microscope (LSM700; Carl Zeiss, Oberkochen, Germany).

**Table 3:** The antibodies used in this study.

Primary antibodies Target	Host species	Dilution	Cat. No.	Vendor
OCT3/4	Mouse	1 : 200	611203	BD
TRA‐1‐60	Mouse	1 : 100	MAB4360	Merck
α‐Synuclein	Mouse	1 : 100	sc‐58480	Santa Cruz Biotechnology
TH	Rabbit	1 : 200	AB152	Merck
TH	Chicken	1 : 100	T9237‐04A	US Biological Life Sciences
ELAVL3/4	Mouse	1 : 100	A21271	Thermo Fisher Scientific
GIRK2	Rabbit	1 : 400	APC‐006	Alomone Labs
Cleaved‐Caspase‐3	Rabbit	1 : 400	9661	Cell Signaling Technology

Secondary antibodies Product name	Dilution	Cat. No.	Vendor
Alexa Fluor 488‐conjugated goat anti‐mouse IgG antibody	1 : 500	A11029	Thermo Fisher Scientific
Alexa Fluor 488‐conjugated goat anti‐mouse IgM antibody	1 : 500	A21042	Thermo Fisher Scientific
Alexa Fluor 568‐conjugated goat anti‐rabbit IgG antibody	1 : 500	A11036	Thermo Fisher Scientific
Alexa Fluor 647‐conjugated goat anti‐chicken IgY antibody	1 : 500	A‐32933	Thermo Fisher Scientific

### Analysis of α‐synuclein accumulation

A confocal microscope (LSM700) was used to obtain z‐stacked fluorescent images of α‐synuclein. The z‐stacked images were then binarized to extract fluorescent signals of α‐synuclein and passed to the mean filter using imagej software (Fiji package; National Institutes of Health, Bethesda, MD, USA) [24, 25]. Accumulated α‐synuclein was defined by the outlying data points based on a boxplot of the clump size derived from healthy hiPSC‐derived neurons (201B7 and 409B2). Total accumulation was calculated as the sum of accumulated α‐synuclein in each image, and it was normalized by the thickness of each z‐stack. The values of total α‐synuclein accumulation are presented as arbitrary units (a.u.). The resulting values from healthy hiPSC‐derived neurons and *PARK4* hiPSC‐derived neurons were compared statistically.

### Evaluation of cleaved‐caspase‐3 in α‐synuclein accumulated cells

Cells exhibiting perinuclear α‐synuclein accumulation were manually identified in fluorescence images obtained by a confocal microscope (LSM700). We then evaluated whether these cells were positive or negative for cleaved‐caspase‐3 (*n* = 81).

### Comparison of the average fluorescence signal intensity of cleaved‐caspase‐3 between 409B2 and PARK4‐4 cells

The average fluorescence signal intensities of cleaved‐caspase‐3 and DAPI were determined from scanned images obtained by confocal microscopy (LSM700). For normalization, the average fluorescence signal intensity of cleaved‐caspase‐3 was divided by the average fluorescence signal intensity of DAPI in each image. The normalized average fluorescence signal intensity was compared between 409B2 and PARK4‐4 cells on day 28 (*n* = 10).

### Antisense oligonucleotide (ASO) treatment

The ASOs used in this study are listed in Table 4. In particular, hSNCA‐121‐AmNA was specific for α‐synuclein mRNA, while the other ASOs (hSNCA‐121‐scr1‐AmNA to hSNCA‐121‐scr4‐AmNA) were scrambled and used as negative controls. Cultures of midbrain DA neurons were fed every other day (from day 14 to 28) with terminal differentiation medium without (untreated control) or with ASOs (final concentration: 3 µm).

**Table 4:** ASOs used in this study.

Label	Name	Target	Sequence^a^
ASO.SNCA	hSNCA‐121‐AmNA	*SNCA*	CTAcatagagaaCAc
ASO.Cont.1	hSNCA‐121‐scr1‐AmNA	—	TACatacgaaacACg
ASO.Cont.2	hSNCA‐121‐scr2‐AmNA	—	AGCataatcgacACa
ASO.Cont.3	hSNCA‐121‐scr3‐AmNA	—	TGCacatgcaaaACa
ASO.Cont.4	hSNCA‐121‐scr4‐AmNA	—	AGCtacatagaaACc

^a^ Capital and small letters represent AmNA and DNA, respectively. All internucleosidic linkages are phosphorothioate linkages.

### Statistical analysis

The statistical significance of two group comparisons was performed using Welch's *t*‐test. The statistical significance of multigroup comparisons was performed using Dunnett's test or two‐way analysis of variance (ANOVA) followed by a *post hoc* multiple comparison test with Benjamini–Hochberg (BH) correction [26]. *P* values of < 0.05, < 0.01, and < 0.001 were considered to indicate statistical significance. Additional details of the methods are presented in the figure legends.

## Results

### Characterization of *PARK4* patient‐derived hiPSCs and healthy donor‐derived hiPSCs for investigation of the dynamics of α‐synuclein in DA neurons

**Fig. 1:**
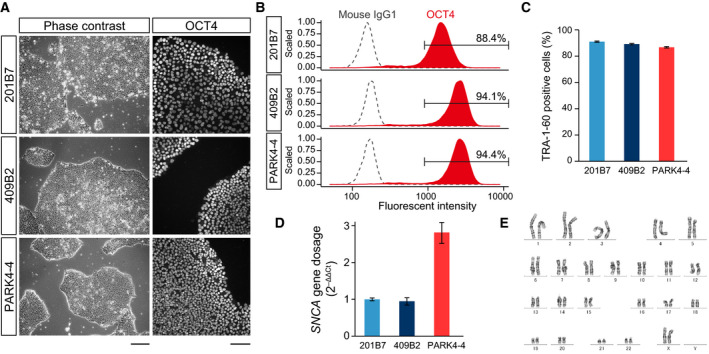
Characterization of *PARK4* patient‐derived hiPSCs and healthy donor‐derived hiPSCs for investigating the dynamics of α‐synuclein in DA neurons. (A) Phase‐contrast images of hiPSC colonies and immunofluorescence images of OCT4‐stained hiPSC colonies. The scale bars indicate 200 and 100 μm for phase‐contrast images and fluorescence images, respectively. (B) Histogram of OCT4 (red) and mouse IgG1 isotype control (black dashed line)‐derived fluorescent intensity obtained from a high‐content screening system. Numbers indicate the populations of OCT4‐positive cells. (C) Population of TRA‐1‐60‐positive cells evaluated by a high‐content screening system. (D) *SNCA* gene copy number analysis using quantitative PCR; $2^{‐\Delta \Delta C_{t}}$ values relative to 201B7 are shown. The error bars represent the standard deviation. (E) Karyotype of PARK4‐4 iPSCs.

To investigate the dynamics of α‐synuclein in DA neurons, which are selectively lost in patients with PD, we established an hiPSC line derived from a *PARK4* patient with *SNCA* triplication (PARK4‐4), which had a typical hiPSC‐like morphology resembling the two healthy donor‐derived hiPSC lines (201B7 and 409B2) (Fig. 1A). These three hiPSC lines were composed of cells with a high population of pluripotent stem cell markers (OCT4 and TRA‐1‐60) just before starting induction of the midbrain floor plate (Fig. 1B,C). We also confirmed that PARK4‐4 cells had an increased copy number of *SNCA* compared to 201B7 and 409B2 cells (Fig. 1D) but had a normal karyotype (Fig. 1E).

### Midbrain floor plate was successfully induced from *PARK4* patient‐derived hiPSCs and healthy donor‐derived hiPSCs

**Fig. 2:**
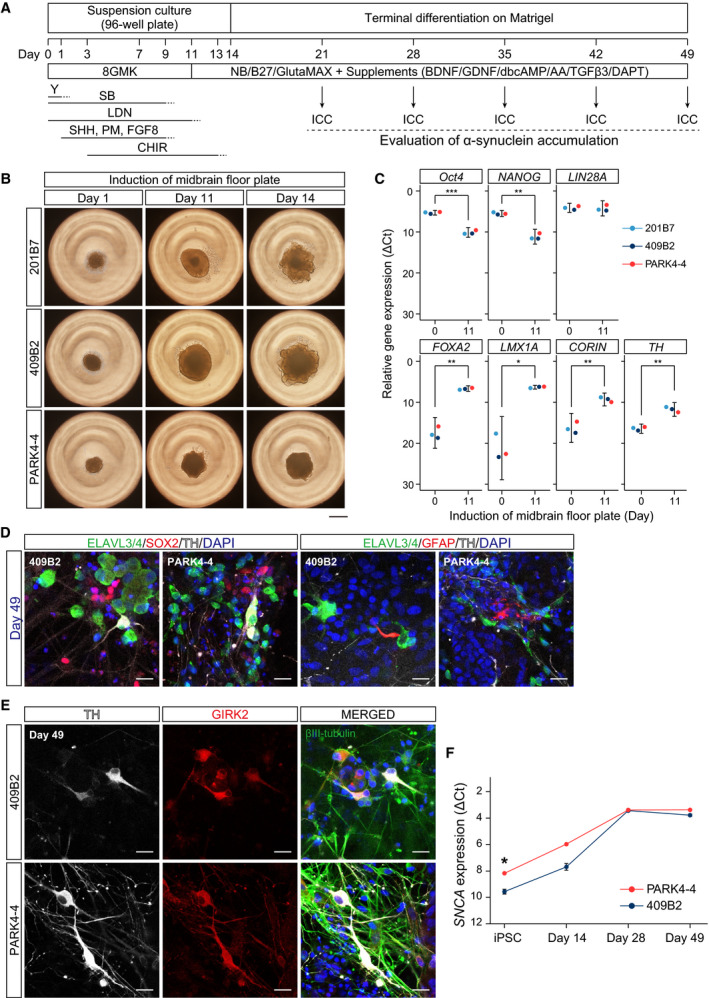
Successful induction of the midbrain floor plate from healthy donor‐derived hiPSCs and *PARK4* patient‐derived hiPSCs. (A) Schematic of midbrain floor plate induction and evaluation of α‐synuclein accumulation during terminal differentiation. (B) Phase‐contrast images of neural aggregates during the induction of the midbrain floor plate (days 1, 11, and 14). The scale bar indicates 500 μm. (C) Relative gene expression levels on days 0 and 11 during the induction of the midbrain floor plate as determined by quantitative PCR (SYBR Green assay). Average ΔC*_t_* values relative to *GAPDH* are shown. Different colors indicate each cell line. The error bars represent the 95% CI. Welch's *t*‐test: *P < 0.05; **P < 0.01; ***P < 0.001. (D) Immunofluorescence images of 409B2 and PARK4‐4 cells on day 49 of differentiation. (left) ELAVL3/4 (green), SOX2 (red), and TH (white) are shown. (right) ELAVL3/4 (green), GFAP (red), and TH (white) are shown. The nuclei were stained with DAPI (blue). The scale bar indicates 20 μm. (E) Immunofluorescence images of 409B2 and PARK4‐4 cells on day 49 of differentiation. TH (white), GIRK2 (red), and βIII‐tubulin (green) are shown. The nuclei were stained with DAPI (blue). The scale bar indicates 20 μm. (F) Relative *SNCA* expression levels on days 0, 14, 28, and 49 as determined by quantitative PCR (TaqMan Gene Expression assay). Average ΔC*_t_* values relative to *GAPDH* are shown. Different colors indicate each cell line. The error bars represent the standard deviation. Welch's *t*‐test: *P < 0.05.

We performed experiments to efficiently generate midbrain DA neurons from hiPSC lines using previously reported methods with slight modifications [19, 20] (Fig. 2A). *PARK4* patient‐derived hiPSCs (PARK4‐4) and healthy donor‐derived hiPSCs (201B7 and 409B2) were first induced into neural aggregates with properties of the midbrain floor plate for 11 days (Fig. 2B). Thereafter, they continued differentiating to DA neurons for additional 3 days before culturing on Matrigel (Fig. 2B). Quantitative RT‐PCR results showed that pluripotency marker genes—*Oct4* and *NANOG*—were downregulated, whereas the midbrain floor plate marker genes—*FOXA2*, *LMX1A*, and *CORIN*—were upregulated on day 11 of midbrain floor plate induction (Fig. 2C). Although the expression of another pluripotency marker gene—*LIN28A*—was not low, that of the DA neuron marker gene—*TH (tyrosine hydroxylase)*—was slightly high just before terminal differentiation on day 11 (Fig. 2C). The population of TH‐positive DA neuron within pan‐neuronal marker‐positive neurons was almost the same between 409B2 (25.0%, *n* = 108) and PARK4‐4 (21.5%, *n* = 149) (Fig. 2D) on day 49 of differentiation. We also observed some remaining SOX2‐positive undifferentiated cells and GFAP‐positive glial cells in the differentiation culture on day 49 (Fig. 2D). The maturation marker of DA neuron, GIRK2, was observed in both 409B2 and PARK4‐4 cells (Fig. 2E).

### Higher α‐synuclein accumulation in PARK4‐4 cells than in 201B7 and 409B2 cells disappeared with time during terminal differentiation

**Fig. 3:**
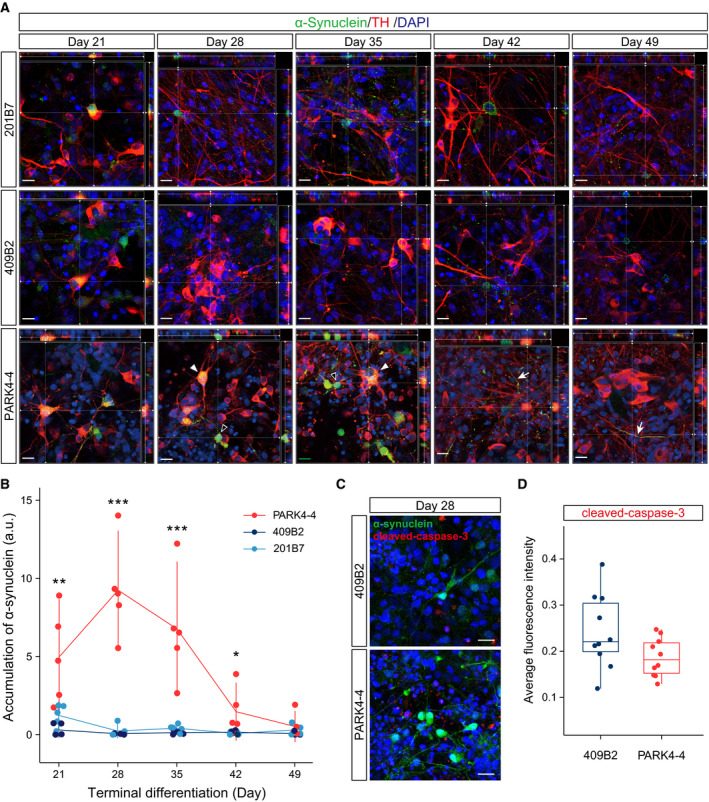
Higher α‐synuclein accumulation in PARK4‐4 cells than in 201B7 and 409B7 cells disappears with time during terminal differentiation. (A) Orthogonal immunofluorescence images of α‐synuclein (green) and TH (red) are shown. The nuclei were stained with DAPI (blue). White arrowheads and open arrowheads indicate strong TH‐positive and TH‐weak/negative cells, respectively. White arrows indicate α‐synuclein‐positive neurites. The scale bar indicates 20 μm. (B) Significant α‐synuclein accumulation was observed in PARK4‐4 cells until day 42. Differences in the accumulation of α‐synuclein protein in each cell line were compared with the accumulation in 201B7 cells on day 21. Dunnett's test: *P < 0.05; **P < 0.01; ***P < 0.001. (C) Immunofluorescence images of 409B2 and PARK4‐4 stained with antibodies specific for α‐synuclein (green) and cleaved‐caspase‐3 (red) on day 28. The nuclei are stained with DAPI (blue). The scale bars represent 20 μm. (D) Average fluorescence intensity of cleaved‐caspase‐3 from 10 images. Different colors indicate each cell line. There was no significant difference (two‐way ANOVA).

Next, we confirmed the expression levels of α‐synuclein on mRNA. We found that the *SNCA* mRNA level was higher in PARK4‐4 than 409B2 cells at the hiPSC stage and on day 14 of differentiation but nearly the same after day 28 (Fig. 2F). We also investigated whether α‐synuclein accumulates only in *PARK4* patient‐derived DA neurons (PARK4‐4) but not in healthy donor‐derived DA neurons (201B7 and 409B2) during terminal differentiation. In addition to neurites, perinuclear α‐synuclein accumulation was readily detected in PARK4‐4, but the accumulation was limited in both 201B7 and 409B2 until day 35 (Fig. 3A). These α‐synuclein accumulations were identified not only in DA neurons with strong TH positivity but also in weakly TH‐positive/TH‐negative cells (Fig. 3A). The total amount of accumulated α‐synuclein was significantly high in PARK4‐4 compared with that in 201B7 on day 21 until day 42 (Fig. 3B). After day 42, however, the level of α‐synuclein accumulation in PARK4‐4 cells decreased to that in 201B7 and 409B2, but accumulation was still observed on some neurites (Fig. 3A,B). To confirm whether perinuclear α‐synuclein accumulation disappeared because of cell death, we immunostained the cells for an apoptosis marker, cleaved‐caspase‐3. A total of 96.3% of cells with perinuclear α‐synuclein accumulations were negative for cleaved‐caspase‐3 for PARK4‐4. We also confirmed that the difference in comparative cleaved‐caspase‐3/DAPI intensity between 409B2 and PARK4‐4 cells was not significant (Fig. 3C,D).

### Treatment with an α‐synuclein‐specific ASO decreased the levels of α‐synuclein mRNA and protein

**Fig. 4:**
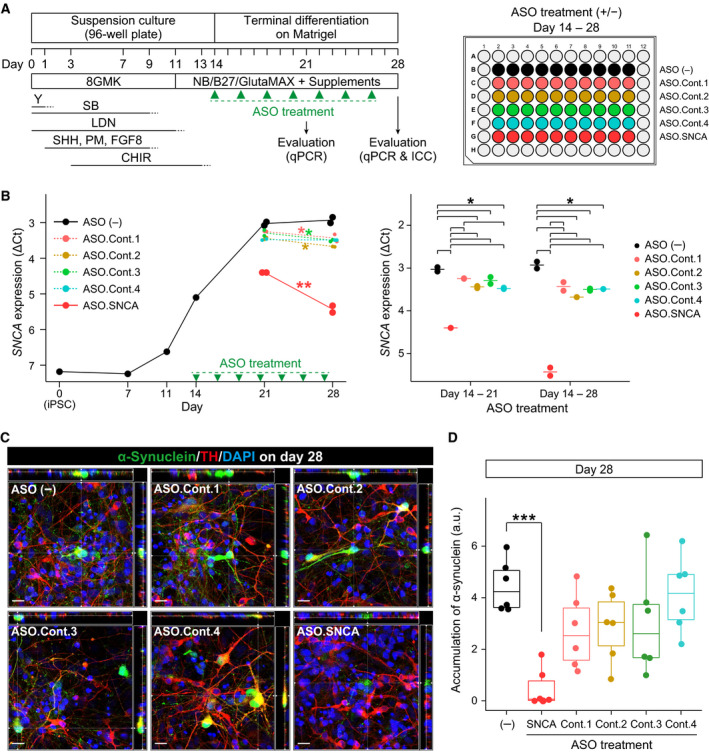
Treatment with an ASO specific for α‐synuclein decreased the levels of α‐synuclein mRNA and protein. (A) Schematic of the induction of midbrain DA neurons and treatment with ASOs. (B) Relative expression levels of *SNCA* gene during the induction of midbrain DA neurons as analyzed by quantitative PCR (TaqMan Gene Expression assay). The left panel shows a comparison of *SNCA* gene expression levels between days 21 and 28. The right panel shows the difference in *SNCA* expression levels among treatments at day 21 or 28. The average ΔC*_t_* values relative to *GAPDH* are shown. Different colors indicate each ASO treatment. Two‐way ANOVA followed by a *post hoc* multiple comparison test with BH correction: *P < 0.05; **P < 0.01. (C) Orthogonal immunofluorescence images of α‐synuclein (green) and TH (red). The nuclei are stained with DAPI (blue). The scale bars represent 20 μm. (D) Treatment with an ASO specific for α‐synuclein significantly reduced α‐synuclein accumulation on day 28. The α‐synuclein protein accumulation levels were compared between cells treated with ASOs and those not treated with ASOs [ASO (−)]. Dunnett's test: ***, *P* < 0.001.

Finally, we investigated whether the use of a recently developed ASO specific for α‐synuclein mRNA (*SNCA*) [8] can decrease the levels of α‐synuclein in PARK4‐4 cells. Cultures of midbrain DA neurons were fed every other day with terminal differentiation medium without ASOs (untreated control; ASO (−)) or with media supplemented with ASOs (an *SNCA*‐specific or scrambled sequences as negative controls; Fig. 4A) from days 14 to 28. *SNCA* mRNA expression increased gradually during terminal differentiation in the cells from days 14 to 21 and then significantly decreased in cells treated with an *SNCA*‐specific ASO as well as negative control ASOs from days 21 to 28 (Fig. 4B, left). On both days 21 and 28 (after 7‐ and 14‐day ASO treatment), *SNCA* mRNA expression levels decreased further in cells treated with an *SNCA*‐specific ASO than in those treated with negative control ASOs or without *SNCA* silencing [ASO (−)] (Fig. 4B, right). The results of immunofluorescence analysis showed that α‐synuclein accumulation obviously appeared in ASO (−) cells and in cells treated with negative control ASOs, but accumulation was extremely limited in cells treated with *SNCA*‐specific ASO (Fig. 4C). Although the total amount of accumulated α‐synuclein tended to be lower in cells treated with negative control ASOs than with ASO (−), similar to the results of gene expression analysis (Fig. 4B), the levels were significantly low in cells treated with *SNCA*‐specific ASO (Fig. 4D).

## Discussion

Parkinson's disease is a major neurodegenerative disease wherein the pathological symptoms are manifested later in life [1, 2]. Although causative genes have been identified for familial forms of PD [3, 4], they remain unknown in ~ 90% cases of sporadic PD [5]. Among the pathological genomic loci of PD, *PARK4*—which involves *SNCA* duplication or triplication without pathological mutations—has been identified [11]. As α‐synuclein is a major component of LBs, a histological hallmark of PD in addition to loss of DA neurons in the SNc, larger amounts of α‐synuclein in the brain are not favorable. In fact, symptom onset in *PARK4* patients occurs at earlier ages, for example, 34 years on average [11, 27], even without any pathological mutation in the α‐synuclein gene. Moreover, numerous genetic variations associated with the sporadic form of PD have been identified based on genome‐wide association studies [28]. A recent report showed that the transcriptional deregulation of *SNCA* is caused by a common PD‐associated risk variant located in the noncoding distal enhancer element of *SNCA* [29]. Since *PARK4* patients, as well as those with the sporadic form of PD, do not express neurological symptoms before PD onset, we hypothesized that a similar mechanism of α‐synuclein accumulation might be underway in both PD types. In other words, the mechanisms accurately regulating α‐synuclein levels before symptom onset might be similar in both types of PD. Thus, newer therapies for sporadic PD could be developed by investigating *PARK4* patients because it is reasonable to consider that these α‐synuclein regulation mechanisms may be functional at higher levels in DA neurons derived from *PARK4* patients with *SNCA* triplication or duplication [30].

In this study, we investigated α‐synuclein dynamics during development in DA neurons differentiated from a PARK4‐4 hiPSC line (derived from a *PARK4* patient with *SNCA* triplication) and 201B7 and 409B2 hiPSC lines (derived from a healthy donor) (Fig. 1A–E) [13, 15, 16]. To induce differentiation to midbrain DA neurons, which are selectively lost in patients with PD, we applied previously developed methods comprising two stages: midbrain floor plate induction and terminal differentiation [19, 20] (Fig. 2A). Midbrain floor plates were successfully generated (Fig. 2B,C), and DA neurons were efficiently produced during the terminal differentiation stage not only from 201B7 and 409B2 cells but also from PARK4‐4 cells (Figs 2D,E and 3A). A maturation marker of DA neurons, GIRK2, was detected, along with some GFAP‐positive glial cells (Fig. 2D,E), under our culture conditions.

The *SNCA* mRNA level was higher in PARK4‐4 cells than in 409B2 cells at the hiPSC stage and on day 14 of differentiation but almost the same on day 49 of differentiation (Fig. 2F). Therefore, α‐synuclein was increased in both healthy donor‐derived cells and *PARK4* patient‐derived cells during neuronal differentiation. Interestingly, however, the levels of α‐synuclein accumulation at the perinucleus and neurites in PARK4‐4 cells were significantly higher than those in 201B7 and 409B2 cells until day 42 of terminal differentiation (Fig. 3A,B). This accumulation of α‐synuclein is consistent with that described previously [30, 31, 32]. Although we considered that we reproduced the pathology of PD, α‐synuclein accumulation decreased to levels observed in 201B7 and 409B2 cells by day 49 of differentiation (Fig. 3A,B). Therefore, we hypothesized that this obliteration of α‐synuclein accumulation was the consequence of perinuclear α‐synuclein accumulation‐induced cellular apoptosis. However, only a few cells with α‐synuclein accumulation exhibited immunopositivity for cleaved‐caspase‐3, an apoptotic marker, clearly indicating that α‐synuclein accumulation did not significantly induce cell death during terminal differentiation (Fig. 3C,D). Because we did not detect signals for phosphorylated Ser 129 of α‐synuclein (data not shown), we considered that this accumulated α‐synuclein itself may not be a pathological species. As hiPSC‐derived neurons are considered immature [33], α‐synuclein accumulation may be appropriately regulated in the early developmental stage even in neurons with *SNCA* triplication. Otherwise, we might need a longer culture system or some unrevealed factors to induce pathological aggregation of accumulated α‐synuclein. Together, these findings indicated the difficulty of reproducing the full phenotype of late‐onset neuronal degenerative disease like PD within a limited short *in vitro* culture time, and these issues should be addressed in future studies.

Although the mechanisms underlying α‐synuclein accumulation in LBs remain unknown, therapies aimed at decreasing α‐synuclein levels show potential as therapeutic strategies, and several systems have already been developed for this purpose such as *SNCA*‐specific ASOs [8, 9] and α‐synuclein‐specific antibodies [10]. We investigated whether a previously developed *SNCA*‐specific ASO [8] effectively prevents α‐synuclein accumulation in PARK4‐4‐derived DA neurons (Fig. 4A). Because α‐synuclein accumulation was obliterated with time during differentiation, the effect of ASOs was analyzed from days 14 to 28 of terminal differentiation (Fig. 4A). The *SNCA*‐specific ASO could downregulate *SNCA* mRNA (Fig. 4B) and α‐synuclein protein expression (Fig. 4C). Therefore, *SNCA*‐specific ASOs may be a feasible chemoprophylactic approach in patients with familial or sporadic PD.

One of the study limitations is that we only obtained snapshots of α‐synuclein accumulation in cells during terminal differentiation, and some cells with α‐synuclein accumulation were weakly positive or negative in terms of staining for TH. We could not determine whether these cells express TH at later time points or eventually died. Therefore, further studies are needed to elucidate how cells with α‐synuclein accumulation remain alive using techniques such as time‐lapse imaging.

## Conclusions

Midbrain DA neurons, differentiated from *PARK4* patient‐derived hiPSCs with *SNCA* triplication, showed a significant amount of α‐synuclein accumulation at the perinucleus and neurites compared with that of DA neurons differentiated from healthy donor‐derived hiPSCs. However, these α‐synuclein accumulations disappeared with time during their terminal differentiation. This phenomenon might further elucidate the mechanism underlying the regulation of α‐synuclein levels in *PARK4* patients, who are healthy even before the earlier onset of PD, and also in sporadic PD patients who account for ~ 90% of all PD cases.

## Conflict of interest

The authors declare no conflict of interest.

## Author contributions

HF conceived of and designed the experiments, performed the experiments, analyzed the data, contributed the reagents/materials/analysis tools, wrote the paper, prepared the figures and/or tables, and reviewed drafts of the paper. KT conceived of and designed the experiments, contributed the reagents/materials/analysis tools, and reviewed drafts of the paper. MS contributed reagents/materials/analysis tools and reviewed drafts of the paper. MN conceived of and designed the experiments, contributed reagents/materials/analysis tools, prepared the figures and/or tables, and reviewed drafts of the paper. SO conceived of and designed the experiments, contributed reagents/materials/analysis tools, prepared the figures and/or tables, and reviewed drafts of the paper. KB conceived of and designed the experiments, contributed reagents/materials/analysis tools, and reviewed drafts of the paper. TS conceived of and designed the experiments, contributed reagents/materials/analysis tools, and reviewed drafts of the paper. DI conceived of and designed the experiments, contributed reagents/materials/analysis tools, and reviewed drafts of the paper. HO conceived of and designed the experiments, contributed reagents/materials/analysis tools, and reviewed drafts of the paper. HM conceived of and designed the experiments, wrote the paper, and reviewed drafts of the paper. YK conceived of and designed the experiments, wrote the paper, and reviewed drafts of the paper.

## Data Availability

All data generated during this study are included in this article.
